# Effects of Acute Heat Stress on a Newly Established Chicken Hepatocyte—Nonparenchymal Cell Co-Culture Model

**DOI:** 10.3390/ani10030409

**Published:** 2020-03-01

**Authors:** Máté Mackei, Andor Molnár, Szabolcs Nagy, László Pál, Csaba Kővágó, Péter Gálfi, Károly Dublecz, Ferenc Husvéth, Zsuzsanna Neogrády, Gábor Mátis

**Affiliations:** 1 Department of Physiology and Biochemistry, Division of Biochemistry, University of Veterinary Medicine, István utca 2, H-1078 Budapest, Hungary; Neogrady.Zsuzsanna@univet.hu (Z.N.); Matis.Gabor@univet.hu (G.M.); 2 Department of Animal Science, Georgikon Faculty, University of Pannonia, Deák Ferenc utca 16, H-8360 Keszthely, Hungary; andor.molnar@georgikon.hu (A.M.); nagy.szabolcs@georgikon.hu (S.N.); pal-l@georgikon.hu (L.P.); dublecz@georgikon.hu (K.D.); hferenc48@georgikon.hu (F.H.); 3 Department of Pharmacology and Toxicology, University of Veterinary Medicine, István utca 2, H-1078 Budapest, Hungary; kovago.csaba@univet.hu (C.K.); galfi.peter@univet.hu (P.G.)

**Keywords:** heat stress, liver, cell culture, heat shock proteins, pro-inflammatory cytokines, oxidative stress, broiler chicken

## Abstract

**Simple Summary:**

Among many environmental stress factors, heat stress can intensely deteriorate animal health, welfare, and productivity in poultry farming. In the present study, a novel cell culture comprised of different liver cell types from chickens was established to serve as a model for studying the effects of acute heat stress. Short, 1 h heat exposure strongly affected liver cells by increasing metabolism, triggering oxidative stress, and decreasing the generation of certain mediatory molecules of the cellular stress response. However, these alterations were normalized after 2 h of heat stress, suggesting a fast adaptation of liver cells. The results of this study underline the impact of short-term heat stress as a potential harmful factor affecting liver function in chickens.

**Abstract:**

Heat stress is one of the most important issues in broiler flocks impairing animal health and productivity. On a cellular level, excess heat exposure can trigger heat shock response acting for the restoration of cell homeostasis by several mechanisms, such as affecting heat shock protein synthesis, redox homeostasis and pro-inflammatory cytokine production. The major aim of this study was to establish a novel avian hepatocyte—nonparenchymal cell co-culture as a model for investigating the cellular effects of heat stress and its interaction with inflammation in chicken liver. Cell fractions were isolated by differential centrifugation from a freshly perfused chicken liver, and hepatocyte mono-cultures as well as hepatocyte–nonparenchymal cell co-cultures (with cell ratio 6:1, hepatocytes to nonparenchymal cells, mimicking a milder hepatic inflammation) were prepared. Isolated and cultured cells were characterized by flow cytometry and immunocytochemistry applying hepatocyte- and macrophage-specific antibodies. Confluent cell cultures were exposed to 43 °C temperature for 1 or 2 h, while controls were cultured at 38.5 °C. The metabolic activity, LDH enzyme activity, reactive oxygen species (H_2_O_2_) production, extracellular concentration of heat shock protein 70 (HSP70), and that of the pro-inflammatory cytokines interleukin (IL-)6 and IL-8 were assessed. Shorter heat stress applied for 1 h could strongly influence liver cell function by significantly increasing catabolic metabolism and extracellular H_2_O_2_ release, and by significantly decreasing HSP70, IL-6, and IL-8 production on both cell culture models. However, all these alterations were restored after 2 h heat exposure, indicating a fast recovery of liver cells. Hepatocyte mono-cultures and hepatocyte—nonparenchymal cell co-cultures responded to heat stress in a similar manner, but the higher metabolic rate of co-cultured cells may have contributed to a better capability of inflamed liver cells for accommodation to stress conditions. In conclusion, the established new primary cell culture models provide suitable tools for studying the hepatic inflammatory and stress response. The results of this study highlight the impact of short-term heat stress on the liver in chickens, underline the mediatory role of oxidative stress in acute stress response, and suggest a fast cellular adaptation potential in liver cells.

## 1. Introduction

Environmental stressors have highly relevant negative impacts on the health condition and productivity of the chickens in broiler farming. Several technological and nutritional problems may increase stress and contribute to immunomodulation, such as overstocking, excess dust, wet litter, improper or contaminated feed, and heat stress. Among the numerous concerns, heat stress is an important issue in intensive poultry production, with increasing significance due to the climate change. Elevated environmental temperature, especially when combined with higher air humidity, can cause suffering and remarkably deteriorates the health, welfare, and growth performance of broilers [1].

On cellular level, a specific stress response pathway, called heat shock response (HSR) is initiated by heat stress acting for the restoration of cell homeostasis by complex alterations of several signaling and metabolic pathways [2]. Heat shock proteins (HSPs) are primarily involved in the successful cellular adaptation to stress conditions, while the synthesis of most other proteins gets discontinued during HSR [3]. Further, HSR is usually linked to enhanced oxidative stress, reflected by elevated levels of pro-oxidants (such as reactive oxygen species, ROS) and inadequate level of antioxidants [1]. Heat-triggered oxidative stress of broilers resulted in enhanced lipid peroxidation in the liver and the heart muscle [4], and increased oxidative damage of muscular proteins due to the dysfunction of the respiratory chain [5,6]. Among various tissues, liver was found highly susceptible to heat-provoked oxidative stress in broilers [4].

Heat stress was also reported to cause functional changes in the immune response by altering the gene expression of pro-inflammatory cytokines, such as increasing splenic interleukin (IL-)4 and IL-12 concentrations in chickens [7]. Further, the cellular immune system may also get diminished by heat stress, reflected by decreased total white blood cell count [8] and macrophage activity [9]. Heat stress also strongly affected the immune response by decreasing the splenic IL-6 and IL-12, and further, caecal IL-1β and IL-10 gene expression in *Salmonella* Enteritidis infected chickens [10].

Based on the aforementioned data, heat-associated distress of the liver, due to its central role in the metabolism of nutrients and xenobiotics, may be critical for the whole organism by destructing the maintenance of metabolic health. In addition to hepatocytes, Kupffer cells as the resident liver macrophages, together with further circulation-derived macrophage cells, are predominantly involved in hepatic inflammatory and stress response [11]. Further, these cells also play a key role in the regulation of metabolic processes, serving as a link between inflammation and metabolism [12]. Therefore, monitoring the function of hepatic nonparenchymal (NP) cells, primarily macrophages in the complex regulation of inflammation and stress response could highlight new ways of improving animal health and productivity.

To study the effects of heat stress on the function of different liver cells in chickens, novel hepatic cell culture models were aimed to be developed. Our research group has already established and characterized a primary co-culture comprised of hepatocytes and NP cells (mostly Kupffer cells) of pig origin, which can serve as a proper tool for investigations on the cellular inflammatory and stress response [13]. Since no similar avian liver cell culture models have been prepared yet, the first main goal of the present study was to develop a hepatic parenchymal—NP cell co-culture from chickens. Due to the difference in size of hepatic cells in birds and mammals, cell isolation procedures had to be adapted to chickens, and separated cell fractions needed to be characterized. Further, the molecular effects of a shorter (1 h) and a longer (2 h) heat exposure on the oxidative status, HSP70 and pro-inflammatory cytokine production were aimed to be assessed on the newly established primary liver cell cultures. Applying mono-cultures of hepatocytes and co-cultures of parenchymal and NP cells may highlight the role of different cell types in stress response. The established co-culture as an inflammatory model can presumably contribute to understand the link between hepatic inflammation and distress.

## 2. Materials and Methods

### 2.1. Cell Isolation and Culturing Conditions

Liver cells were freshly isolated from three-week-old male broiler chickens of the Ross-308 strain reared and fed according to the Ross technology [14], and obtained from Gallus Ltd. (Devecser, Hungary). For setting up the cell isolation and separation procedure, some preliminary studies were carried out using one broiler in each trial (eight totally). For the characterization of cell fractions gained by the finally established method (with immunocytochemistry and flow cytometry) and to study the cellular effect of heat stress, liver cells had to be isolated from the same single chicken in order to ensure the homogeneity of the prepared primary cell cultures. All experimental procedures with the animals were carried out in accordance with the national and EU laws, as well as with the institutional guidelines, and were confirmed by the Local Animal Welfare Committee of the University of Veterinary Medicine, Budapest (permission number: PEI/001/1430-4/2015, approval date: 27 April 2015). All chemicals were purchased from Sigma-Aldrich (Darmstadt, Germany) except when otherwise specified.

The animals were slaughtered in carbon dioxide narcosis by decapitation, and the liver was perfused via the gastropancreaticoduodenal vein of the hepatic portal system. All perfusion buffers were previously warmed up to 40 °C and were freshly oxygenated with Carbogen (95% O_2_, 5% CO_2_); the velocity of the perfusion was set to 30 mL/min. In the first stage of the multi-step perfusion, 150 mL ethylene glycol bis (2-aminoethyl ether) tetraacetic acid (EGTA, 0.5 mM) containing Hanks’ balanced salt solution (HBSS) buffer (previously supplemented with 0.035% NaHCO_3_) was applied, followed by 150 mL EGTA-free HBSS. In the final step, 100 mL HBSS buffer, freshly supplemented with 100 mg collagenase type IV (Nordmark, Uetersen, Germany), 7 mM CaCl_2_, and 7 mM MgCl_2_ was perfused into the liver to disintegrate hepatic parenchymal cells.

After excision of the liver, the capsule was disrupted, and the gained liver cell suspension was filtered through three layers of sterile gauze and was incubated in bovine serum albumin (BSA, 2.5%)—containing HBSS buffer on ice for 45 min to avoid cell aggregate formation. Thereafter, the cell suspension was centrifuged three times at 100× *g* for 3 min, and the hepatocyte-enriched sediment was resuspended in Williams’ Medium E, previously supplemented with 0.22% NaHCO_3_, 50 mg/L gentamycin, 2 mM glutamine, 4 µg/L dexamethasone, 20 IU/L insulin, and 5% foetal bovine serum (FBS).

The NP cell fraction (containing mostly macrophages, primarily Kupffer cells) was separated from the supernatants gained in the low-speed (100× *g*) centrifugation steps. The supernatants were centrifuged at 350× *g* for 10 min to sediment the remaining hepatocytes, cell detritus and red blood cells, and the newly gained supernatant was centrifuged again at 800× *g* for 10 min. The final sediment, containing NP cells, was also resuspended in Williams’ Medium E. The viability of hepatocytes and NP cells was confirmed by the trypan blue exclusion test, and cell yield was examined by cell counting in Bürker’s chamber to adjust the appropriate cell concentrations (hepatocyte mono-cultures: 10^6^ cells/mL; co-cultures: 8.5 × 10^5^ cells/mL hepatocytes; 1.5 × 10^5^ cells/mL NP cells).

All cell cultures were prepared on 6-well, 96-well (Greiner Bio-One, Frickenhausen, Germany), and lumox x-well (Sarstedt, Nümbrecht, Germany) cell culture dishes, previously coated with collagen type I according to the manufacturer’s instructions. The NP cells were seeded at first, and after their rapid attachment to the plate surface in 20 min, to prepare hepatocyte—NP cell co-cultures, the culture medium was removed and hepatocytes were seeded in the cell ratio of 6:1 (hepatocyte to NP cells). Hepatocyte mono-cultures were also prepared by seeding hepatocyte-enriched fraction onto cell culture dishes. The seeding volume was 1.5 mL/well on 6-well plates, 100 µL/well on 96-well plates, and 300 µL/well on lumox x-well dishes. All cell cultures were incubated at 38.5 °C in humid atmosphere with 5% CO_2_. Culture media were changed 4 h after seeding, and confluent monolayers were gained following 24 h culturing.

### 2.2. Characterizing Cell Cultures with Giemsa Staining and Immunocytochemistry

To confirm cell morphology, 48-h-cultured confluent monolayers on 6-well plates were stained with Giemsa, and to assess the presence and the ratio of various liver cell types in different cell culture models, immunocytochemical analyses were applied with chicken specific antibodies. Albumin was detected with a chicken specific, fluorescein isothiocyanate (FITC) coupled anti-albumin antibody (Cedarlane, Burlington, Canada). The macrophages (mainly Kupffer cells as the resident liver macrophages) in NP cell fractions were labelled by using a chicken macrophage specific phycoerythrin (PE) coupled antibody (Southern Biotech, Uden, The Netherlands).

Isolated cells and cell cultures (following 48 h culturing) on lumox x-well plates were fixed in phosphate buffered saline (PBS) containing 4% formaldehyde for 30 min at room temperature (21 °C). After rinsing the fixed cells in PBS (3 times 5 min), they were permeabilized with Triton-X (0.25%) containing PBS for 20 min, and were subsequently blocked in PBS supplemented with 5% goat serum, 3% BSA, and 0.1% Triton-X for 60 min at room temperature. Antibodies were dissolved (in the ratio of 1:100 for anti-albumin and 1:50 for macrophage specific antibody) in PBS containing 1% BSA and were applied overnight at 4 °C. After counterstaining with 4’,6-diamidino-2-phenylindole (DAPI), cell cultures were analyzed with an Olympus CKX-41-type fluorescent microscope and a Canon EOS 1100D camera.

### 2.3. Characterizing Cell Fractions with Flow Cytometry

Hepatocyte enriched and NP cell containing fractions were examined with flow cytometry. Cell suspensions with approximately 1 × 10^6^/mL cell concentration were filtered prior to data acquisition with Sysmex CellTrics filters (30 µm, Ref No. 04-0042-2316), then subsequently analyzed with a Beckman Coulter FC 500 flow cytometer equipped with an air-cooled 20 mW, 488 nm Argon ion laser. Forward and side scatter values were recorded in the corresponding photodetectors. The flow rate was set to “low” (10 µL/min). A total of 10,000 events were collected per sample.

Data analysis was done with Flowing free software (version 2.5.1, www.flowingsoftware.com) and FCS Express 7 Plus (version 7.00.0037, www.denovosoftware.com) by drawing two-dimensional plots, showing forward (FS) versus side scatter (SS). Both parameters were displayed on log axes.

### 2.4. Treatments and Measurements of Cellular Metabolic Activity, LDH Activity, H_2_O_2_, HSP70, IL-6, and IL-8 Concentrations

After 24 h culturing, culture media were changed to fresh FBS-free Williams’ Medium E, and confluent mono- and co-cultures on 6-well and 96-well plates were incubated at 43 °C for 1 or 2 h to mimic heat stress, while control cells were incubated further at 38.5 °C. The incubation conditions were set based on literature data and our pilot studies, considering that birds have higher physiological body temperature than mammals; however, cells isolated from avian species are often cultured at temperatures similar to mammalian cells. Normal incubation temperatures of avian cell cultures are ranging from 37 to 41.5 °C, and temperatures mimicking heat stress are varied between 40 and 45 °C [15,16,17,18], hence, as a compromise, 38.5 °C was chosen for maintaining control cells, and 43 °C for studying acute heat stress.

Following heat exposure, the metabolic activity of cells on 96-well plates was monitored by the CCK-8 assay according to the manufacturer’s instructions, detecting the amount of NADH+H^+^ gained in the catabolic pathways. Briefly, 10 µL CCK-8 reagent and 100 µL fresh Williams’ Medium E were given to the cultured cells, and the absorbance was measured at 450 nm with a Multiskan GO 3.2 reader after 2 h incubation at 38.5 °C. In order to monitor cytotoxicity, extracellular lactate dehydrogenase (LDH) activity was measured by a specific photometric assay (Diagnosticum Ltd., Budapest, Hungary). First, 200 µL working reagent (containing 56 mM phosphate buffer, pH = 7.5; 1.6 mM pyruvate, and 240 µM NADH+H^+^) was mixed with a 10 µL cell culture medium. The enzyme activity was assessed by a kinetic method, measuring the absorbance of samples at 340 nm with a Multiskan GO 3.2 reader.

Culture media of 6-well plates were collected directly after the applied treatments for measuring extracellular ROS (H_2_O_2_), HSP70, IL-6, and IL-8 concentrations. Following the removal of media, cultured cells from both 6-well and 96-well plates were gently washed in PBS and were subsequently lysed in a M-PER buffer supplemented with 1% Halt Protease Inhibitor Cocktail and 1% ethylene diamine tetraacetic acid (EDTA) (Thermo Fisher Scientific, Waltham, MA, USA) for assaying total protein concentrations. All culture media and cell lysate samples were stored at −80 °C until further processing.

The H_2_O_2_ concentration of cell supernatants was assessed with the Amplex Red Hydrogen Peroxide Assay Kit (Thermo Fisher Scientific, Waltham, MA, USA). The applied substrate (Amplex Red) reacts with H_2_O_2_ in a horseradish peroxidase (HRP) catalyzed reaction, producing highly fluorescent resorufin. After 30 min incubation of 50 µL culture media with 50 µL freshly prepared, Amplex Red (100 µM) and HRP (0.2 U/mL) containing a working solution at room temperature, fluorescence was detected with a Victor X2 2030 fluorometer (λ_ex_ = 560 nm; λe_m_ = 590 nm). The concentrations of HSP70, IL-6, and IL-8 were measured in the culture media of 6-well dishes by chicken specific ELISA kits (Cat. No. MBS734158, MBS268769, and MBS013823, respectively; MyBioSource, San Diego, CA, USA) following the manufacturer’s instructions. The absorbance values were quantified at 450 nm with a Multiskan GO 3.2 reader. Total protein concentration of cell lysates was assessed with the Pierce^TM^ Bicinchoninic Acid (BCA) Protein Assay (Thermo Fisher Scientific, Waltham, MA, USA) as indicated by the manufacturer, applying BSA as a standard, adding 25 µL sample to 200 µL reagent mixture and measuring the absorbance after 30 min incubation at 37 °C at 562 nm with a Multiskan GO 3.2 reader.

### 2.5. Statistics

All treatments were applied in triplicates on 6-well plates, and six wells were included in each group on 96-well plates. Data of metabolic activity, LDH enzyme activity, H_2_O_2_, HSP70, IL-6, and IL-8 concentrations of cell culture supernatants were all standardized to the total protein concentrations of cell lysates. Data were analyzed with one-way ANOVA and with Tukey’s post-hoc test, applying the R 2.14.0 software. All results are expressed as mean ± SEM, the level of significance was set at *p* < 0.05.

## 3. Results

### 3.1. Characterizing Cell Cultures with Giemsa Staining and Immunocytochemistry

**Figure 1:**
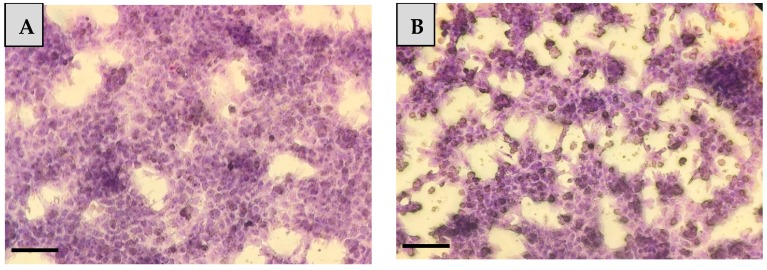
Giemsa staining of **(A)** hepatocyte mono-cultures and **(B)** hepatocyte—nonparenchymal cell co-cultures after 48 h culturing (20× magnification, bar = 100 µm).

In our study, after staining confluent hepatocyte mono-cultures and hepatocyte—NP cell co-cultures with Giemsa, typical morphological features of parenchymal and NP liver cells could be observed (Figure 1).

**Figure 2:**
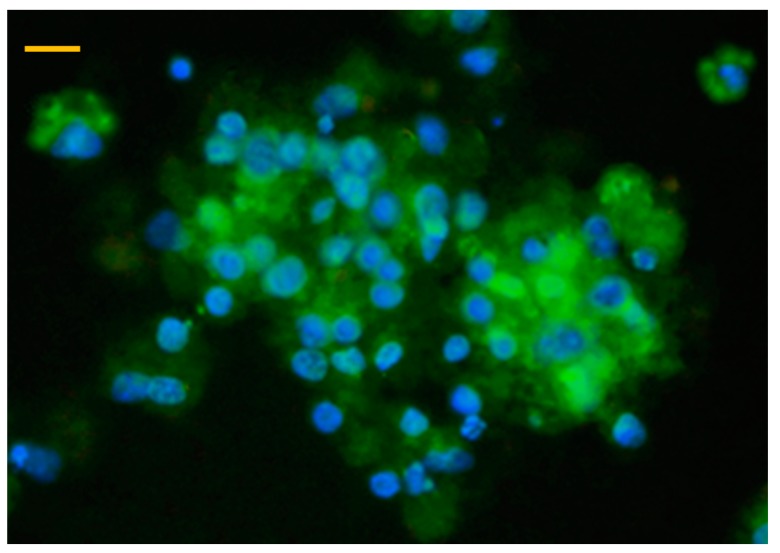
Immunofluorescent detection of hepatocytes in a hepatocyte—nonparenchymal cell co-culture after 48 h culturing with a chicken specific, fluorescein isothiocyanate (FITC) coupled anti-albumin antibody (40× magnification, bar = 40 µm). Blue colour indicates cell nuclei with DAPI staining, while green colour refers to hepatocytes detected with the FITC conjugated antibody.

**Figure 3:**
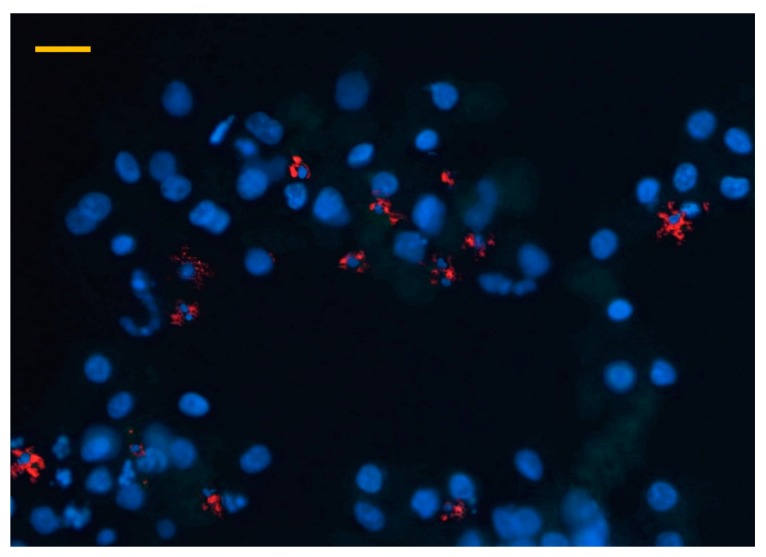
Immunofluorescent detection of macrophages in a hepatocyte—nonparenchymal cell co-culture after 48 h culturing with a phycoerythrin (PE) coupled chicken macrophage specific antibody (40× magnification, bar = 40 µm). Blue colour indicates cell nuclei with DAPI staining, while red colour refers to macrophages detected with the PE conjugated antibody.

Immunocytochemical detection of albumin revealed that cultured parenchymal cells showed strong positivity indicated by the green fluorescence of the applied FITC coupled anti-albumin antibody, confirming the presence of hepatocytes in both freshly isolated cell suspensions and cells after 48 h culturing (Figure 2). In addition, the majority of isolated and cultured NP cells were positively stained by the macrophage specific PE conjugated antibody, reflected by the red fluorescence (Figure 3).

### 3.2. Characterizing Cell Fractions with Flow Cytometry

**Figure 4:**
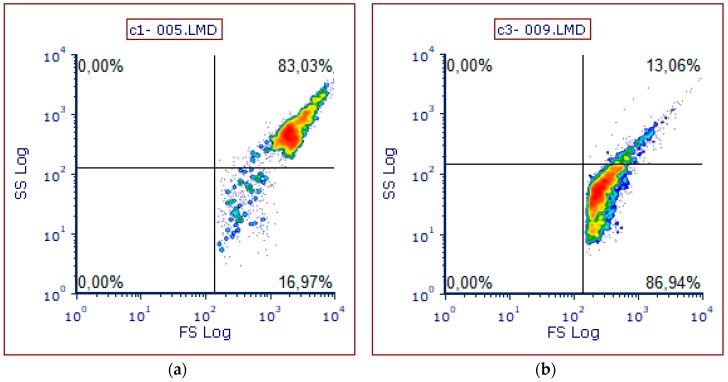
A representative density plot figure showing the forward (FS) and side (SS) scatter profiles of hepatocytes (**a**) and nonparenchymal cells (**b**).

Applying scatter profiles to monitor the characteristics and homogeneity of the separated cell suspensions, two well-defined, reproducible fractions were isolated (Figure 4). The type of cells comprising these fractions was confirmed with immunofluorescent staining of isolated and cultured cells as described above, using FITC coupled anti-albumin and PE conjugated macrophage specific antibodies.

### 3.3. Measurements of Cellular Metabolic Activity, LDH Activity, H_2_O_2_, HSP70, IL-6, and IL-8 Concentrations

**Figure 5:**
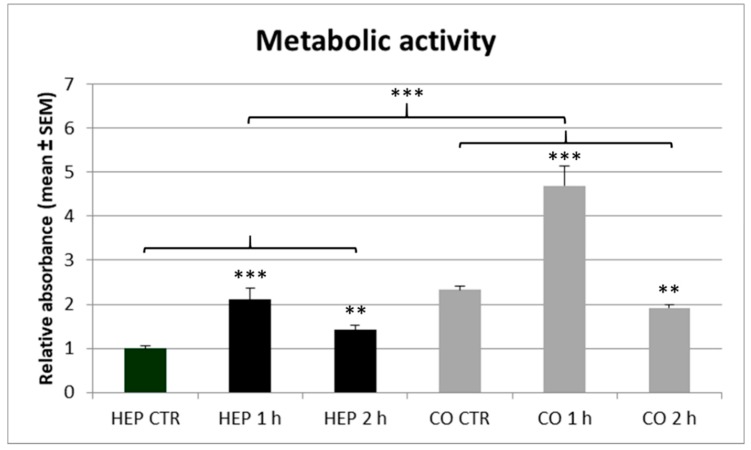
The metabolic activity of hepatocyte mono-cultures (“HEP”) and hepatocyte—nonparenchymal cell co-cultures (“CO”) as indicated by the CCK-8 assay. The “CTR” refers to control cells with no heat exposure, while “1 h” and “2 h” indicate incubation of cell cultures at 43 °C for 1 or 2 h, respectively. Results are expressed as mean ± standard error of the mean (SEM), *n* = 6/group. Asterisks over bars of “1 h” and “2 h” refer to significant differences compared to “CTR” cells within the same cell culture model. Significant differences between cell culture models are indicated with asterisks on the clamps. ** *p* < 0.01, *** *p* < 0.001.

The metabolic activity of cultured cells, monitored with the CCK-8 assay (Figure 5), was higher in co-cultures compared to hepatocyte mono-cultures, independently from the heat exposure (*p* < 0.001). The shorter, 1 h lasting heat stress increased the catabolic activity of hepatocyte mono-cultures and hepatocyte—NP cell co-cultures compared to controls (*p* < 0.001). The longer, 2 h heat exposure elevated the metabolic activity of hepatocyte mono-cultures (*p* = 0.006), but with a lower extent than the 1 h heat stress, whereas it reduced metabolic activity of co-cultured cells (*p* = 0.004).

**Figure 6:**
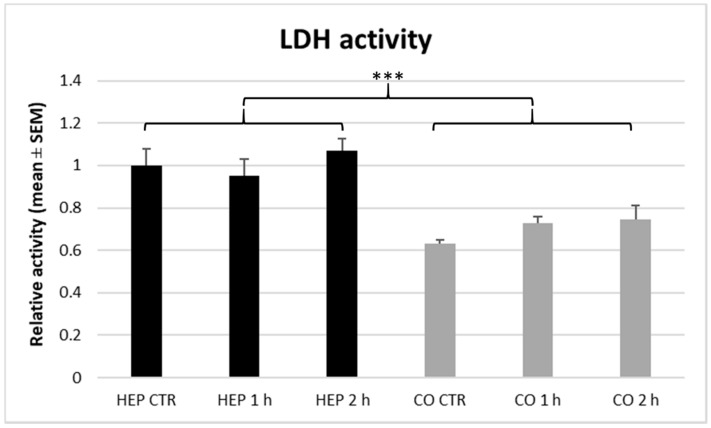
The lactate dehydrogenase (LDH) activity of hepatocyte mono-cultures (“HEP”) and hepatocyte—nonparenchymal cell co-cultures (“CO”) as indicated by specific photometric assay. The “CTR” refers to control cells with no heat exposure, while “1 h” and “2 h” indicate incubation of cell cultures at 43 °C for 1 h or 2 h, respectively. Relative activities were calculated by considering the mean value of control hepatocyte mono-cultures as 1. Results are expressed as mean ± standard error of the mean (SEM), *n* = 3/group. Asterisks over bars of “1 h” and “2 h” refer to significant differences compared to “CTR” cells within the same cell culture model. Significant differences between cell culture models are indicated with asterisks on the clamps. *** *p* < 0.001.

The extracellular LDH activity indicating the amount of necrotic cells is shown in Figure 6. According to our results, LDH activity was not affected by heat exposure on both cell culture models. In comparison with the mono-cultured hepatocytes, significantly lower (*p* < 0.001) LDH activity was detected in case of co-cultures independently from the heat stress.

**Figure 7:**
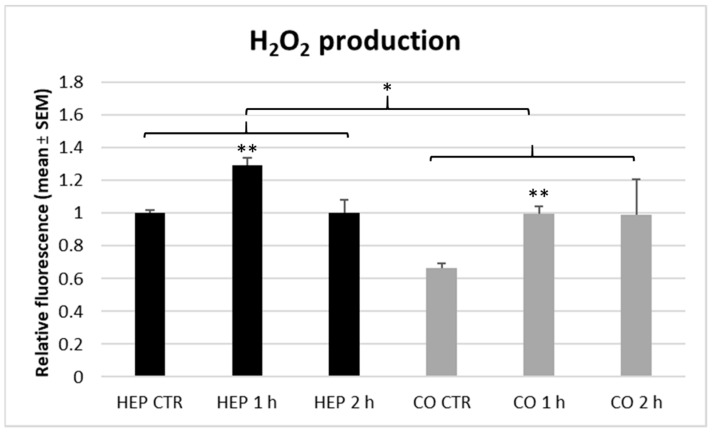
The hydrogen peroxide (H_2_O_2_) production of hepatocyte mono-cultures (“HEP”) and hepatocyte—nonparenchymal cell co-cultures (“CO”) as indicated by the Amplex Red assay. The “CTR” refers to control cells with no heat exposure, while “1 h” and “2 h” indicate incubation of cell cultures at 43 °C for 1 or 2 h, respectively. Relative fluorescences were calculated by considering the mean value of control hepatocyte mono-cultures as 1. Results are expressed as mean ± standard error of the mean (SEM), *n* = 3/group. Asterisks over bars of “1 h” and “2 h” refer to significant differences compared to “CTR” cells within the same cell culture model. Significant differences between cell culture models are indicated with asterisks on the clamps. * *p* < 0.05, ** *p* < 0.01.

The extracellular ROS production of cell cultures was investigated by measuring the H_2_O_2_ concentration of culture media with the Amplex Red assay (Figure 7). When comparing hepatocyte mono-cultures and hepatocyte—NP co-cultures, slightly, but significantly lower extracellular ROS concentration was detected in the latter case (*p* = 0.048). The shorter term (1 h) heat exposure increased the ROS release of hepatocyte mono-cultures (*p* = 0.004) and hepatocyte—NP cell co-cultures (*p* = 0.003) compared to controls, while the longer (2 h) heat stress did not influence the H_2_O_2_ concentration of cell supernatants on both cell culture models.

**Figure 8:**
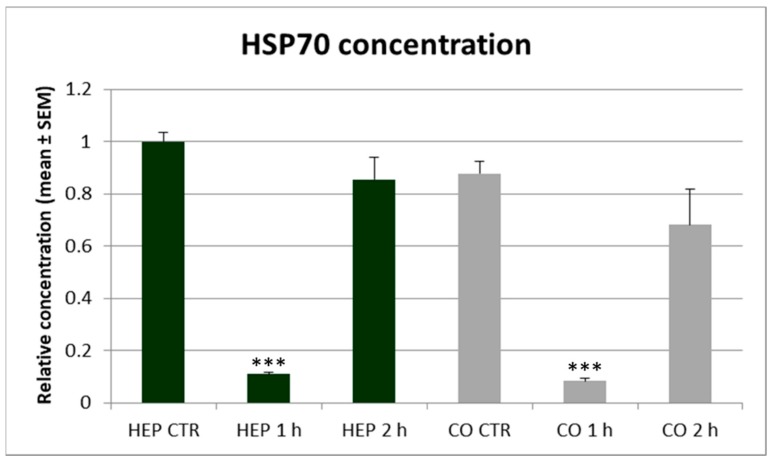
The heat shock protein 70 (HSP70) concentration in culture media of hepatocyte mono-cultures (“HEP”) and hepatocyte—nonparenchymal cell co-cultures (“CO”) as measured by a chicken specific ELISA assay. The “CTR” refers to control cells with no heat exposure, while “1 h” and “2 h” indicate incubation of cell cultures at 43 °C for 1 or 2 h, respectively. Relative concentrations were calculated by considering the mean value of control hepatocyte mono-cultures as 1. Results are expressed as mean ± standard error of the mean (SEM), *n* = 3/group. Asterisks over bars of “1 h” and “2 h” refer to significant differences compared to “CTR” cells within the same cell culture model. *** *p* < 0.001.

The HSP70 concentration of cell culture supernatants (Figure 8), measured by a specific ELISA assay, did not differ significantly on different cell culture models. The shorter (1 h) heat exposure strongly decreased the HSP70 release of both hepatocyte mono-cultures and hepatocyte—NP cell co-cultures (*p* < 0.001 in both cases), while the HSP70 level was normalized after 2 h heat stress as no significant difference could be found in the HSP70 concentrations of control and 2 h long heat exposed cells in any cell cultures.

**Figure 9:**
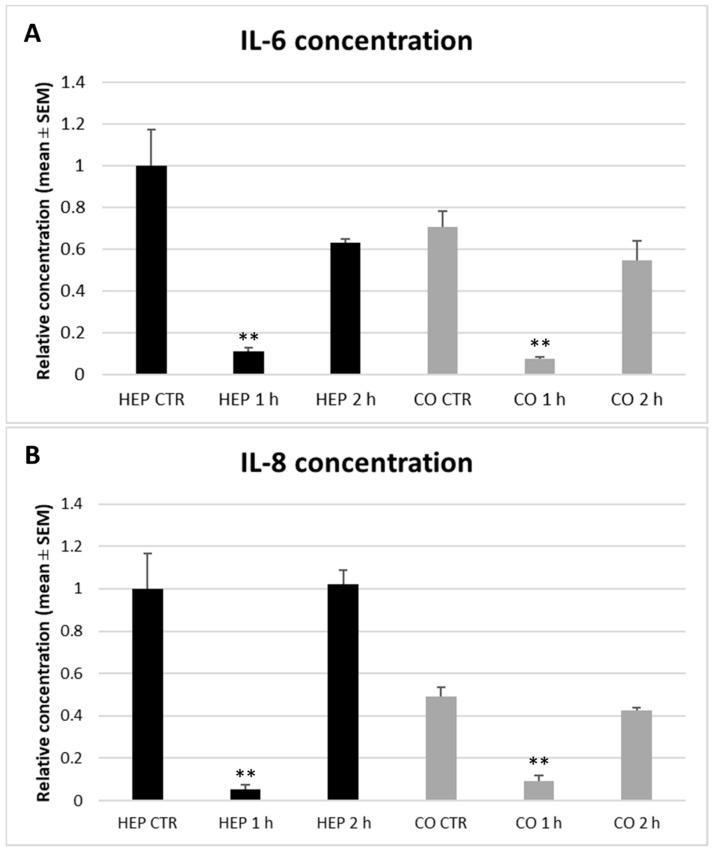
Interleukin-6 (IL-6, A) and interleukin-8 (IL-8, B) concentration in culture media of hepatocyte mono-cultures (“HEP”) and hepatocyte—nonparenchymal cell co-cultures (“CO”) detected by a chicken specific ELISA assay. The “CTR” refers to control cells with no heat exposure, while “1 h” and “2 h” indicate incubation of cell cultures at 43 °C for 1 or 2 h, respectively. Relative concentrations were calculated by considering the mean value of control hepatocyte mono-cultures as 1. Results are expressed as mean ± standard error of the mean (SEM), *n* = 3/group. Asterisks over bars of “1 h” and “2 h” refer to significant differences compared to “CTR” cells within the same cell culture model. ** *p* < 0.01.

Similarly to HSP70, the concentration of pro-inflammatory cytokines IL-6 and IL-8 showed no significant differences between hepatocyte mono-cultures and co-cultures in culture media as measured by specific ELISA kits (Figure 9A,B). A strong decrease in both IL-6 and IL-8 levels was detected after 1 h heat exposure on both hepatocyte mono-cultures and hepatocyte—NP cell co-cultures compared to controls (IL-6: *p* = 0.007 and *p* = 0.001, IL-8: *p* = 0.005 and *p* = 0.001, respectively). Following the longer, 2 h heat stress, interleukin concentrations tended to return to the baseline values as no significant differences were observed when comparing control and 2 h heat exposed cells.

## 4. Discussion

In the present study novel primary hepatic cell culture models have been successfully established from chickens. Based on the investigation of the separated cell fractions with flow cytometry and on the immunofluorescent characterization of cultured cells, hepatocyte mono-cultures and hepatocyte—NP cell co-cultures have been prepared from chicken liver. As justified by immunocytochemistry, the NP cell fraction comprised of mainly macrophages, first of all Kupffer cells as the resident liver macrophages and presumably also circulation-derived macrophages; however, the presence of other NP cell types (such as stellate cells or biliary endothelial cells) can be also suggested.

A monolayer hepatocyte—Kupffer cell and a double-layered enterohepatic co-culture have been also established recently by our research group from swine [13,19], but to the best of our knowledge, no similar avian cell cultures were available until now. From chickens, mainly tumorigenic cell lines, such as the Chicken Hepatocellular Carcinoma Cell Line (indicated as LMH cells) [20,21] or embryonic liver cell cultures [22] were used. The newly prepared chicken cell cultures enable studies concerning the specific role of parenchymal and NP cells as the main liver cell fraction, and the hepatocyte—NP cell co-culture model can mimic various inflammatory states by setting different cell type ratios. The applied ratio of 6:1 (hepatocytes to NP cells) refers to a milder hepatic inflammation with moderate intrahepatic macrophage migration [13]. On this co-culture, the interaction of the inflammatory and stress response can be studied, including molecular alterations of cell function, such as the pro- and anti-inflammatory cytokine production and the redox homeostasis of the cultured liver cells. The main advantage of these models is that they are nontumorigenic primary cell cultures, hence the results can be better extrapolated to the in vivo conditions of the healthy or inflamed chicken liver.

The other major goal of the present study was to investigate the cellular effects of acute heat stress on the newly established cell culture models. The applied heat exposure and incubation conditions were set based on previously available literature data and our pilot studies. In spite of the higher physiological body temperature of birds, avian cells are often cultured at relatively lower temperatures, similarly to mammalian cultured cells. For instance, the aforementioned hepatic LMH cells, chicken embryonic fibroblast (CEF) cells, or chicken primary myocardial cells were cultured at 37 °C [15,16,17], while a chicken macrophage-like cell line was maintained at 41.5 °C [18]. Similarly, different protocols do exist for the in vitro modelling of heat stress. An elevation of the ambient temperature from 37 to 43 °C could trigger maximal heat stress response in LMH cells [15,23], while CEF cells were heat stressed by increasing temperature from 37 to 40–44 °C [16]. Further, heat stress response was evoked in myocardial cells by elevating temperature from 37 to 42 °C [17], and heat stress was modelled in chicken macrophage-like cells by incubating cells at 45 °C in contrast to control cells cultured at 41.5 °C [18]. The time course of heat exposure was also different in various studies; the incubation time mostly ranged between 1 and 5 h to provoke short or medium-term heat stress [15,17,18]. Based on the parameters of these avian in vitro experiments, considering also the characteristics and the morphology of primary liver cells (continuously monitored by phase contrast microscopy), in our study control cells were incubated at 38.5 °C and heat exposed cell cultures at 43 °C for 1 and 2 h to mimic the cellular effects of intense, shorter, and longer heat stress.

According to our results, heat stress strongly influenced the metabolic activity, redox state, HSP70, and pro-inflammatory cytokine production of cultured chicken liver cells. The short-term, 1 h heat exposure remarkably increased the catabolic activity of both hepatocyte mono-cultures and hepatocyte—NP cell co-cultures, with a higher extent in the latter case. Heat-associated enhanced metabolic rate may contribute to a better accommodation of cells to the increased temperature, which may be reflected in the alleviation of metabolic activity after 2 h of heat stress. On hepatocyte mono-cultures, cellular metabolic activity was still increased after the longer heat exposure compared to controls, but with a lower extent; however, on co-cultures it was already slightly lower after 2 h of heat treatment than in control cells. These results suggest a time-dependent metabolic adaptation to heat stress, hence cultured liver cells tended to recover after the longer heat exposure, also reflected in the other parameters measured, such as HSP70, IL-6, and IL-8 concentrations of cell supernatants. The heat-triggered changes in the metabolic activity of mono-cultured hepatocytes and co-cultured parenchymal and NP cells showed a similar pattern, but co-cultures seemed to accommodate faster to the altered temperature conditions than hepatocyte mono-cultures. Based on our findings, extracellular LDH activity was not affected by heat stress indicating that the applied heat exposures were not cytotoxic and did not induce necrosis of the cultured cells. Compared to the hepatocyte mono-cultures, the observed lower LDH activity of co-cultures may relate to the higher metabolic activity of these cultures.

Numerous studies reported that heat stress is commonly associated with increased oxidative stress caused by elevated ROS production or inadequate amount of antioxidants [1]. In comparison with other organs, liver was found the most sensitive to heat-triggered oxidative stress response in chickens [4]. In the present study, 1 h lasting heat stress increased the H_2_O_2_ concentration of cell supernatants on both cell culture models, indicating an elevation in hepatocellular ROS release and presumably contributing to increased oxidative stress in the liver. Similarly to the metabolic activity, extracellular ROS levels tended to be normalized after 2 h of heat exposure. When comparing hepatocyte mono-cultures with hepatocyte—NP co-cultures, both models responded in the same manner as no significant differences could be found in the H_2_O_2_ concentration of culture media gained from different cell cultures.

The concentrations of HSP70, IL-6, and IL-8 in culture media were altered similarly by heat exposure. After the shorter (1 h) heat treatment, the level of extracellular HSP70 and those of the measured pro-inflammatory cytokines were intensively decreased, with an average extent of approx. 90% on both hepatocyte mono-cultures and hepatocyte—NP cell co-cultures. However, all these concentrations tended to be normalized following the longer, 2 h heat exposure. A similar heat stress associated decline in the HSP70 level was reported in a previous study with rat myocardial cells, where the intracytoplasmic HSP70 concentration was reduced by a short term, 1 h long heat exposure, whereas it was restored after 2 h heat incubation [24]. It can be suggested that the utilization of heat-shock proteins in an intense short term heat stress exceeded their synthesis, resulting in decreased HSP70 levels, but after 2 h, cells could produce a sufficient amount of HSP70 to fulfill the increased requirements and contribute to the restoration of physiological cell function, reflected by normalized metabolic activity and ROS levels. Intracellular HSP70 protein expression was gradually increased by heat stress of 1 to 5 h in another in vitro study done on chicken myocardial cells [21]. In addition, a 2 h long heat stress induced the expression of the HSPA2 gene encoding HSP70 on a chicken macrophage-like cell line [18].

The effects of heat stress on the immune response have been described in certain studies, but limited data is available concerning heat-triggered changes in hepatic immune function. Heat stress could provide controversial immunomodulatory action on the pro-inflammatory cytokine production as it stimulated splenic IL-4 and IL-12 [7], but decreased splenic IL-6, IL-12 gene expression, and also that of IL-1β and IL-10 in caecal tonsils of chickens [10]. However, it should be underlined that these data were gained after chronic in vivo heat stress. The latter immunosuppressive actions were found only in *Salmonella* Enteritidis infected chickens, reflecting a possible interaction of infection with inflammatory and stress response [10]. Heat stress can also suppress cellular immunity by reducing total white blood cell count and macrophage activity [8,9]. In the present study, 1 h of heat stress downregulated the hepatic production of both measured pro-inflammatory cytokines, IL-6 and IL-8, but their levels returned to the baseline after 2 h of heat exposure.

The connection of HSPs and inflammatory mediators has been already studied by analyzing the liver transcriptome of chickens [25]. Most HSPs, such as HSP70, were upregulated by a 3 h long heat stress, while the expression of pro-inflammatory cytokines was mostly low and not affected by heat exposure [25]. However, it can be suggested that HSPs possess a key regulatory role in mitigating immune and inflammatory response under heat stress [26]. In our present cell culture study, HSP70 and pro-inflammatory cytokines responded to heat stress in a similar manner as they were downregulated after 1 h, but normalized within 2 h of heat exposure.

Based on our results and in line with previous studies [25], it can be suggested that the potential impact of a shorter heat stress on liver cell function is much higher than that of a longer heat exposure. Both 1 and 2 h incubation at 43 °C can be considered as acute heat stress, but there were significant differences between the shorter and longer heat exposure. The 1 h lasting intense heat stress could provoke dramatic changes, such as stimulating catabolic metabolism and ROS release and strongly decreasing HSP70 and pro-inflammatory cytokine production, but the majority of these alterations could be mitigated within 2 h of exposure by successful cellular adaptation. Hence, the critical role of short term heat stress in broiler farming has to be emphasized as it can impair liver function and the health of chickens.

When comparing the applied different cell culture models, mostly similar results were received on hepatocyte mono-cultures and hepatocyte—NP cell co-cultures. In case of metabolic activity, the co-culture serving as an inflammatory model showed higher baseline level, which was altered by heat stress to the same extent as on mono-cultured hepatocytes. However, it can be suggested that the metabolic rate of co-cultures was normalized faster as already a moderate decrease was observable after 2 h of heat stress compared to controls. These results indicate an increasing action of inflammation (mimicked by including mostly macrophages as NP cells in co-cultures) on cell metabolic rate and suggest a better capability of inflamed liver cells for accommodation to short term acute heat stress. This is consistent with the observed lower baseline level of extracellular ROS concentration in the hepatocyte—NP cell co-cultures compared to the hepatocyte monoculture.

In conclusion, the established novel primary cell culture models provide useful tools for studying the inflammatory and stress response in the liver of chickens. The successful separation of hepatocytes and NP cells and the preparation of a hepatocyte—NP cell co-culture from chickens enable investigations on the role of different cell types and on the interaction of stress and hepatic immune response. According to the 3R principle, it is also noteworthy, that using the above described and newly established primary cell culture models, considerable amount of data can be achieved using only one chicken, instead of setting up a complete in vivo study applying a large number of animals. Based on our results, a short term (1 h) intense heat stress can largely influence liver cell functions by increasing metabolism and extracellular H_2_O_2_ release, and by decreasing HSP70, IL-6, and IL-8 production. However, all these alterations were restored after 2 h of heat exposure, indicating a fast recruitment of liver cells. These data highlight the great impact of short term heat stress on the functions of chicken liver cells, underline the mediatory role of oxidative stress in acute stress response and imply a fast cellular adaptation potential of liver cells.

## References

[1] Surai P.F., Kochish I.I. (2017). Antioxidant Systems and Vitagenes in Poultry Biology: Heat Shock Proteins. Heat Shock Proteins.

[2] Richter K., Haslbeck M., Buchner J. (2010). The Heat Shock Response: Life on the Verge of Death. Mol. Cell.

[3] Gupta S.C., Sharma A., Mishra M., Mishra R.K., Chowdhuri D.K. (2010). Heat Shock Proteins in Toxicology: How Close and How Far?. Life Sci..

[4] Lin H., Decuypere E., Buyse J. (2006). Acute Heat Stress Induces Oxidative Stress in Broiler Chickens. Comp. Biochem. Physiol. Part A Mol. Integr. Physiol..

[5] Punchihewage Don A., Attuquayefio W., Oh S., Feng X., Ahn D., Min B. (2018). PSIX-2 Effects of Early Heat Conditioning on Heat Stress-Induced Changes in Broiler Growth Performance, Oxidative Stress in Blood, and Quality Parameters in Breast Meat. J. Anim. Sci..

[6] Huang C., Jiao H., Song Z., Zhao J., Wang X., Lin H. (2015). Heat Stress Impairs Mitochondria Functions and Induces Oxidative Injury in Broiler Chickens1. J. Anim. Sci..

[7] Ohtsu H., Yamazaki M., Abe H., Murakami H., Toyomizu M. (2015). Heat Stress Modulates Cytokine Gene Expression in the Spleen of Broiler Chickens. J. Poult. Sci..

[8] Mashaly M.M., Hendricks G.L., Kalama M.A., Gehad A.E., Abbas A.O., Patterson P.H. (2004). Effect of Heat Stress on Production Parameters and Immune Responses of Commercial Laying Hens. Poult. Sci..

[9] Bartlett J.R., Smith M.O. (2003). Effects of Different Levels of Zinc on the Performance and Immunocompetence of Broilers under Heat Stress. Poult. Sci..

[10] Quinteiro-Filho W.M., Calefi A.S., Cruz D.S.G., Aloia T.P.A., Zager A., Astolfi-Ferreira C.S., Piantino Ferreira J.A., Sharif S., Palermo-Neto J. (2017). Heat Stress Decreases Expression of the Cytokines, Avian β-Defensins 4 and 6 and Toll-like Receptor 2 in Broiler Chickens Infected with Salmonella Enteritidis. Vet. Immunol. Immunopathol..

[11] Kolios G. (2006). Role of Kupffer Cells in the Pathogenesis of Liver Disease. World J. Gastroenterol..

[12] Baffy G. (2009). Kupffer Cells in Non-Alcoholic Fatty Liver Disease: The Emerging View. J. Hepatol..

[13] Mátis G., Kulcsár A., Petrilla J., Talapka P., Neogrády Z. (2016). Porcine Hepatocyte-Kupffer Cell Co-Culture as Anin Vitromodel for Testing the Efficacy of Anti-Inflammatory Substances. J. Anim. Physiol. Anim. Nutr..

[14] Aviagen (2018). Ross Broiler Management Handbook.

[15] Sun L., Lamont S.J., Cooksey A.M., McCarthy F., Tudor C.O., Vijay-Shanker K., DeRita R.M., Rothschild M., Ashwell C., Persia M.E. (2015). Transcriptome Response to Heat Stress in a Chicken Hepatocellular Carcinoma Cell Line. Cell Stress Chaperones.

[16] Ibtisham F., Zhao Y., Nawab A., Liguang H., Wu J., Xiao M., Zhao Z., An L. (2018). The Effect of High Temperature on Viability, Proliferation, Apoptosis and Anti-Oxidant Status of Chicken Embryonic Fibroblast Cells. Braz. J. Poult. Sci..

[17] Xu J., Tang S., Song E., Yin B., Bao E. (2017). Inhibition of Heat Shock Protein 70 Intensifies Heat-Stressed Damage and Apoptosis of Chicken Primary Myocardial Cells in Vitro. Mol. Med. Rep..

[18] Slawinska A., Hsieh J.C., Schmidt C.J., Lamont S.J. (2016). Heat Stress and Lipopolysaccharide Stimulation of Chicken Macrophage-Like Cell Line Activates Expression of Distinct Sets of Genes. PloS ONE.

[19] Paszti-Gere E., Matis G., Farkas O., Kulcsar A., Palocz O., Csiko G., Neogrady Z., Galfi P. (2013). The Effects of Intestinal LPS Exposure on Inflammatory Responses in a Porcine Enterohepatic Co-Culture System. Inflammation.

[20] Kawaguchi T., Nomura K., Hirayama Y., Kitagawa T. (1987). Establishment and Characterization of a Chicken Hepatocellular Carcinoma Cell Line, LMH. Cancer Res..

[21] Amin A., Nöbauer K., Patzl M., Berger E., Hess M., Bilic I. (2012). Cysteine Peptidases, Secreted by Trichomonas Gallinae, Are Involved in the Cytopathogenic Effects on a Permanent Chicken Liver Cell Culture. PloS ONE.

[22] Kumar R., Chandra R., Shukla S.K. (2003). Isolation of etiological agent of hydropericardium syndrome in chicken embryo liver cell culture and its serological characterization. Indian J. Exp. Biol..

[23] Gabis K.K., Gildemeister O.S., Pepe J.A., Lambrecht R.W., Bonkovsky H.L. (1996). Induction of Heme Oxygenase-1 in LMH Cells. Comparison of LMH Cells to Primary Cultures of Chick Embryo Liver Cells. Biochim. Et Biophys. Acta.

[24] Chen H.B., Zhang X.C., Cheng Y.F., Abdelnasir A., Tang S., Kemper N., Hartung J., Bao E.D. (2015). Association of Heat Shock Protein 70 Expression with Rat Myocardial Cell Damage during Heat Stress in Vitro and in Vivo. Genet. Mol. Res..

[25] Lan X., Hsieh J.C.F., Schmidt C.J., Zhu Q., Lamont S.J. (2016). Liver Transcriptome Response to Hyperthermic Stress in Three Distinct Chicken Lines. BMC Genom..

[26] Pockley A.G. (2003). Heat Shock Proteins as Regulators of the Immune Response. Lancet.

